# Reconciling the Patient's Role in the Improvement of Health Outcomes: Medical Informatics' Newest Frontier

**DOI:** 10.2196/jmir.6.2.e14

**Published:** 2004-05-20

**Authors:** Warren J Winkelman

**Affiliations:** ^1^Centre for Global eHealth InnovationUniversity Health NetworkToronto ONCanada

In this issue of the Journal of Medical Internet Research, the reader is presented with two important studies that focus on the challenges of integrating patient participation and partnership in medical informatics. Both studies address the enormous potential of information technology to effect change in health by influencing patient behavior.

The study by Ross et al is a randomized controlled trial of SPPARO (System Providing Patients Access to Records Online), a patient-accessible electronic patient records (EPR) system implemented at the University of Colorado, measuring its impact on health outcomes and patient satisfaction [1]. SPPARO is one of a handful of organizational EPR patient-access projects with a substantial body of peer-reviewed literature easily available for study. SPPARO shares a common identity paradox with these other systems in that it portends to be patient-centered while employing physician-centered design and evaluation frameworks [2]. It is therefore not surprising that, in their study, patient access has little measurable impact on patient-specific health outcomes.

The SPPARO study also serves to illustrate two key dilemmas facing clinical informatics researchers. In defining the unit of analysis, is "access" the antecedent for change in outcomes, or is it more appropriate to look for some kind of behavioral change, like technology acceptance or actual system utilization [3]? Furthermore, in the short time frame which characterize most studies, how realistic is it to expect the substantial, meaningful changes in patient health behavior that could conceivably promote changes in health outcomes [4]?

The second study by Kim and Johnson observes the contributory role of format on the subsequent accuracy of data entry by patients in personal health records (PHR), and vividly illustrates the most important challenge facing developers: how to make the PHR useful for patients [5]. The interfaces reviewed in this paper are presented with little knowledge of the research behind them. As readers, we never really know if these products faced rigorous usability testing or if they were constructed with knowledge or awareness of health literacy. In fact, it appears as if the interfaces were most likely written in physician language. Does a patient's thinking about disease proceed along the same trajectory as a clinician's thinking without substantial training? Or, should a PHR ideally be constructed from the ground up, emphasizing the patient's perception of illness and disease [6]?

In conclusion, medical informatics research must continuously develop the capacity to demonstrate that information technology can effect positive change for patients [7]. These two studies illustrate the importance of availing ourselves of the knowledge gained in other related fields, and applying it to the challenges of our own field. For example, we should familiarize ourselves with validated models for evaluation that have appeared in the social science, behavioral psychology, and information systems literatures in the last several decades, and adapting them to research questions around the relationship between patient behavior, technology use, and health. As we are presently in an age of shrinking healthcare resources and expanding health expectations, the medical informatics academic community has the responsibility to public health decision-makers, healthcare providers, and patients to expeditiously provide high quality evidence for the value of information technology to improve health [8,9].
